# The impact of endometriosis on dietary choices and activities of everyday life: a cross-sectional study

**DOI:** 10.3389/fnut.2023.1273976

**Published:** 2023-09-22

**Authors:** Elisa Mazza, Ersilia Troiano, Santino Mazza, Yvelise Ferro, Antonia Abbinante, Maria Teresa Agneta, Tiziana Montalcini, Arturo Pujia

**Affiliations:** ^1^Department of Medical and Surgical Science, University Magna Græcia, Catanzaro, Italy; ^2^Technical Scientific Association of Food, Nutrition and Dietetics (ASAND), Palermo, Italy; ^3^Clinical Nutrition Unit, University Magna Græcia, Catanzaro, Italy; ^4^Direzione Socio-Educativa, Municipio Roma III Montesacro, Rome, Italy; ^5^Italian Dental Hygienists Association (AIDI), Aosta, Italy; ^6^Complex Operating Unit of Odontostomatology, Department of Interdisciplinary Medicine, Aldo Moro University of Bari, Bari, Italy; ^7^Research Center for the Prevention and Treatment of Metabolic Diseases, University Magna Græcia, Catanzaro, Italy; ^8^Department of Clinical and Experimental Medicine, University Magna Græcia, Catanzaro, Italy

**Keywords:** endometriosis, dietary changes, anti-inflammatory foods, eating habit, everyday life

## Abstract

**Introduction:**

Endometriosis is characterized by ectopic endometrial tissue and severe pain; frequently, women afflicted by this condition resort to non-medical interventions, such as dietary modifications. The aim of this study is to assess the impact of endometriosis on dietary patterns and quality of life.

**Methods:**

An online survey was conducted among Italian women with endometriosis to gather self-reported demographic, clinical, dietary habit, and daily life data post-diagnosis.

**Results:**

A total of 4,078 participants were included. Following an endometriosis diagnosis, 66% reported changes in eating habits, and 92% experienced a decline in daily life. Respondents chose dietary interventions: gluten-free (15%), anti-inflammatory (8%), Mediterranean (7.1%), or ketogenic (4%) diets, to improve health and reduce symptoms. The study revealed a shift in eating habits, with increased consumption of vegetables, fruits (10%), cereals, legumes (6.6%), and fish (4.5%), while reducing dairy products (18.4%), soy-containing foods (6.7%), and high saturated fats (8%). Eating habit changes correlated with endometriosis stages and worsened daily life. Educational level, endometriosis stages, years of symptoms, and eating habit changes linked to changes in daily life.

**Conclusion:**

Our findings emphasize the importance of monitoring eating behaviors to prevent unhealthy habits and malnutrition in women with endometriosis. Further studies are needed to evaluate how different diets impact symptoms and enhance daily life for these individuals.

## 1. Introduction

Endometriosis is the prevalent condition among non-malignant gynecological disorders, impacting approximately 10% of women in their reproductive years. It stands as one of the primary anatomical factors contributing to persistent pelvic pain (1). Endometriosis is commonly characterized as a chronic inflammatory condition that relies on estrogen and involves the presence of endometrium-like tissue functioning outside the uterus. Due to these characteristics, it is now recognized as a systemic disease rather than being confined solely to the pelvic region (1). Indeed, endometriosis frequently coexists with various other conditions including fibromyalgia, migraines, irritable bowel syndrome, mental health disorders, and immunological conditions like rheumatoid arthritis (2). Endometriosis is characterized by various symptoms including chronic pain, dysmenorrhea, dyschezia, dyspareunia, dysuria, fatigue, and reduced fertility. However, the precise causes and mechanisms underlying the development of endometriosis are not fully elucidated at present. The pathogenesis and pathophysiology of this condition remain areas of active research, and further investigations are necessary to achieve a comprehensive understanding of the disease (2, 3). The occurrence of endometriosis involves a complex interplay between endocrine, proinflammatory, and immunological processes (3). The current medical and surgical interventions available for endometriosis frequently prove insufficient in alleviating symptoms (1–3).

The presence of endometriosis can have a profound impact on a woman’s physical and social wellbeing (4, 5), resulting in a significant burden of the disease, both in terms of its economic implications and its effect on quality of life (4). Furthermore, endometriosis leads to increased absenteeism from work or school (6). The condition exerts a significant influence on the mental and emotional wellbeing of women (7), as well as their social activities (8), and sexual relationships (9). Studies have indicated that endometriosis can diminish physical quality of life to a similar extent as experienced by cancer patients (8).

A recent systematic review and meta-analysis demonstrated that endometriosis has a detrimental effect on health-related quality of life comparable to that of chronic pain (10–12). Consequently, many women with endometriosis turn to non-medical methods to manage symptoms and enhance their everyday lives (13). Therefore, women with endometriosis often resort to lifestyle interventions such as rest, heat therapy, meditation, exercise, and dietary changes to manage their symptoms (3).

Dietary interventions, in particular, have shown promising results in improving endometriosis-related symptoms. Studies have indicated that a significant proportion of women with endometriosis (76%) employ self-management strategies, with nearly half of them (44%) opting for dietary changes (14). Another recent study revealed that 55.5% of participants reported that food influenced their endometriosis symptoms, and modifying their diet provided symptom relief (15). Dietary factors may play a role in the progression and development of endometriosis by influencing steroid hormone metabolism, the menstrual cycle, inflammation regulation, oxidative stress, and muscle contraction (16). As a result, diets and dietary modifications adopted by women with endometriosis have garnered increasing attention from researchers. The question of whether and how specific diets and lifestyles can influence the pathogenesis and progression of endometriosis continues to be investigated (16, 17).

The link between dietary factors and endometriosis has gained interest due to the recognition that diet can impact both physiological and pathological processes. Some authors suggest that dietary changes may have therapeutic potential in alleviating chronic inflammatory processes and reducing visceral pain perception (18, 19). Certain natural anti-inflammatory agents, such as Omega-3 polyunsaturated fatty acids (PUFAs) and squalene, a biofunctional lipid compound, have shown beneficial effects on chronic diseases (20–22).

Other diets, such as the Mediterranean Diet, low FODMAP (fermentable oligo-, di-, monosaccharides, and polyols) diet, and gluten-free diet, have also been investigated in relation to chronic inflammatory diseases (23, 24). A recent systematic review focusing on the impact of dietary changes on pain perception in relation to endometriosis demonstrated that diet had a positive influence on pain perception among women with endometriosis. Specifically, a high intake of PUFAs, a gluten-free diet, and a low nickel diet were associated with improved pain management in endometriosis (25) Additionally, adding nutrients with anti-inflammatory and antiestrogenic properties such as antioxidants curcumin, epigallocatechin gallate, quercetin, resveratrol, and inositol, found in fruits, vegetables, and fatty fish, while eliminating pro-inflammatory substances like lactose, saturated fats, and soy, has been suggested to alleviate endometriosis pain (15, 26–29).

Moreover, initial investigations into the effectiveness of probiotics in managing endometriosis in women have shown promising outcomes in terms of pain alleviation (30). Furthermore, studies have reported that the administration of Lactobacillus probiotics can ameliorated endometriosis-associated pain in females (31). Consequently, the existing data implies the significant impact of dietary supplements in inducing favorable alterations in the gut microbiota, which may play a role in promoting human health and lowering the risk of inflammatory conditions, including endometriosis.

Consequently, food choices may have an impact on disease progression and pain perception in endometriosis.

However, it is important to note that robust evidence regarding the relationship between nutrition, a healthy diet, and endometriosis treatment is limited, as are studies exploring the effect of the disease on food choices.

Thus, the aim of this study was to investigate the potential effects of the disease on dietary habits and daily activities in women following an endometriosis diagnosis.

## 2. Materials and methods

The Italian Dental Hygienists Association (AIDI) and the Technical Scientific Association of Food, Nutrition, and Dietetics (ASAND), in collaboration with the Clinical Nutrition Unit of the “Magna Graecia” University of Catanzaro, conducted an online survey to explore various aspects of daily life in women with endometriosis. This cross-sectional study collected data between 9 April and 27 June 2021. An anonymous national survey was administered using the Google Forms tool, targeting women over the age of 18 residing in Italy who self-reported a previous endometriosis diagnosis. Recruitment was carried out through direct survey links and invitations distributed via social media platforms such as Facebook, WhatsApp, Twitter, and Instagram, specifically through AIDI, ASAND, and the Italian Association of Endometriosis. The latter serves as a network to foster discussion, community, and support among women with endometriosis. The study received ethical approval from the Local Ethics Committee in the Calabria Region Central Area (code 355/2021/CE).

### 2.1. Questionnaire

We developed a semi-structured online questionnaire that focused on collecting self-reported sociodemographic data (age, educational level, employment status), the self-perceived impact of endometriosis symptoms on daily life, capturing self-reported disease-related information (years since diagnosis, stage, symptoms, delay in diagnosis, pharmacological treatment, associated autoimmune diseases), and documenting self-reported changes in eating habits following an endometriosis diagnosis (see Supplementary Table S1).

In our study, dietary change was defined as a self-reported modification in one’s previous diet (15, 32–34). The impact of endometriosis symptoms on daily life was investigated through questions related to chronic fatigue, depression and anxiety, sleep disorders, reduced fertility or subfertility, decreased sexual satisfaction, reduced work capacity, diminished social interactions, difficulties in daily activity planning and execution, and pain management challenges (7–12, 35–38). Our questionnaire was developed adapting previous validated tools (7–12, 35–38). Participants provided informed consent via an anonymous online form at the beginning of the questionnaire. Participants were asked to self-report the stage of their endometriosis diagnosis, if known, according to the classification system of the American Society of Reproductive Medicine (ASRM), which includes four stages: Stage I (Minimal), Stage II (Mild), Stage III (Moderate), and Stage IV (Severe) (39). The questionnaire consisted of 31 items, primarily employing closed-ended questions with predefined answer options. Five questions allowed participants to provide open-ended responses and share personal opinions. The survey, on average, required 15–20 min to complete.

To validate our questionnaire, as reported in other studies (40), we conducted a factor analysis using Horn’s parallel analysis for principal components, employing varimax rotation (32, 33, 40). An eigenvalue of 1 was used as a cut-off for determining the number of factors. In total, the explained variance accounted for 64.4% of the variance. To assess internal consistency, reflecting the degree to which the elements of the instrument measure the same construct, we employed Cronbach’s α test. A Cronbach’s α value exceeding 0.70 is indicative of good internal consistency, and our questionnaire achieved a Cronbach’s α value of 0.72.

### 2.2. Statistical analysis

To find a correlation between changes in eating habits and the stage of endometriosis with an *r* value of 0.05, with a study power of 80% and a one-tailed alpha of 0.05, a sample size of 3,134 women with endometriosis was required. Upon closure of the online survey and cessation of data collection, the final database was downloaded as a Microsoft Excel sheet and subjected to data analysis. Open-ended questions were carefully reviewed, condensed, and coded for statistical analysis. Missing data were not imputed during the statistical analysis. The results are presented as absolute (n) and relative (%) frequencies for categorical variables. Pearson’s correlation coefficient was utilized to identify any confounding variables associated with changes in eating habits and daily life, assuming normal distribution for continuous variables. Additionally, a Chi-square test was conducted to examine the changes in eating habits and daily life among participants following the diagnosis of endometriosis, stratified by disease stage. The same test was employed to assess dietary patterns and food choices among women with endometriosis who reported a deterioration in their quality of life. Statistical significance was set at *p* < 0.05 (two-tailed). All statistical analyses were performed using SPSS 25.0 for Windows (IBM Corporation, New York, NY, United States).

## 3. Results

During the survey, a total of 4,078 responses were collected and analyzed. Table 1 presents the demographic and clinical characteristics of women with endometriosis. The largest proportion of participants (45%) fell between the ages of 36 and 45, and 37% of women had a severe stage of the disease. Among the participants, 1,333 were undergoing hormonal treatment at the time of the interview, with 1,331 using oral contraceptive pills or vaginal contraceptive rings (see Table 1). The educational background of the respondents varied, encompassing both secondary school and university education (see Table 1). A delay in diagnosis exceeding 7 years was reported by 39% of the participants (see Table 1). Approximately 17% of women reported having autoimmune diseases, including Sjögren’s syndrome, rheumatoid arthritis, systemic lupus erythematosus, autoimmune thyroid disorder, and others (data not shown). Coeliac disease was reported by 1.2% of the participants.

**Table 1:** Demographic and clinical characteristics of women with endometriosis (*n* = 4,078).

Characteristics		*n* (%)
Age groups	Aged 18–25	332 (8.1)
	Aged 26–35	1,407 (34.5)
	Aged 36–45	1822 (44.7)
	Aged 45 and above	517 (12.7)
Educational level	High school graduate	2040 (50)
	University degree	2038 (50)
ARSM endometriosis stages	Minimal (stage I)	962 (23.6)
	Mild (stage II)	773 (19.0)
	Moderate (stage III)	831 (20.4)
	Severe (stage IV)	1,512 (37.1)
Years of symptoms (years)	< 1	55 (1.3)
	1–3	555 (13.6)
	4–10	1,357 (33.3)
	11–15	700 (17.2)
	16–20	565 (13.9)
	> 20	731 (17.9)
	Did not answer	115 (2.8)
Delay in diagnosis (years)	< 1	1,016 (24.9)
	1–3	877 (21.5)
	4–6	600 (14.7)
	> 7	1,585 (38.9)
Hormonal treatments	Progesterone-like medications	1,333 (32.7)
	Combined oestrogen/progestagen medications	1,331 (32.6)
	Menopause-inducing medications	80 (2.0)
Pain relief medication	Pain killers	237 (5.8)
	NSAIDs	217 (5.3)
	Opioids	23 (0.6)
	Nutritional supplements*	213 (5.2)
	PEA supplements	44 (1.1)

ARSM, American society for reproductive medicine; NSAIDs, non-steroidal anti-inflammatory drugs; PEA, palmitoylethanolamide. *Nutritional supplements (quercetin, curcumin, parthenium, nicotinamide, 5-methyltetrahydrofolate, and omega 3/6).

### 3.1. Changes in eating habits after the diagnosis of endometriosis

**Figure 1:**
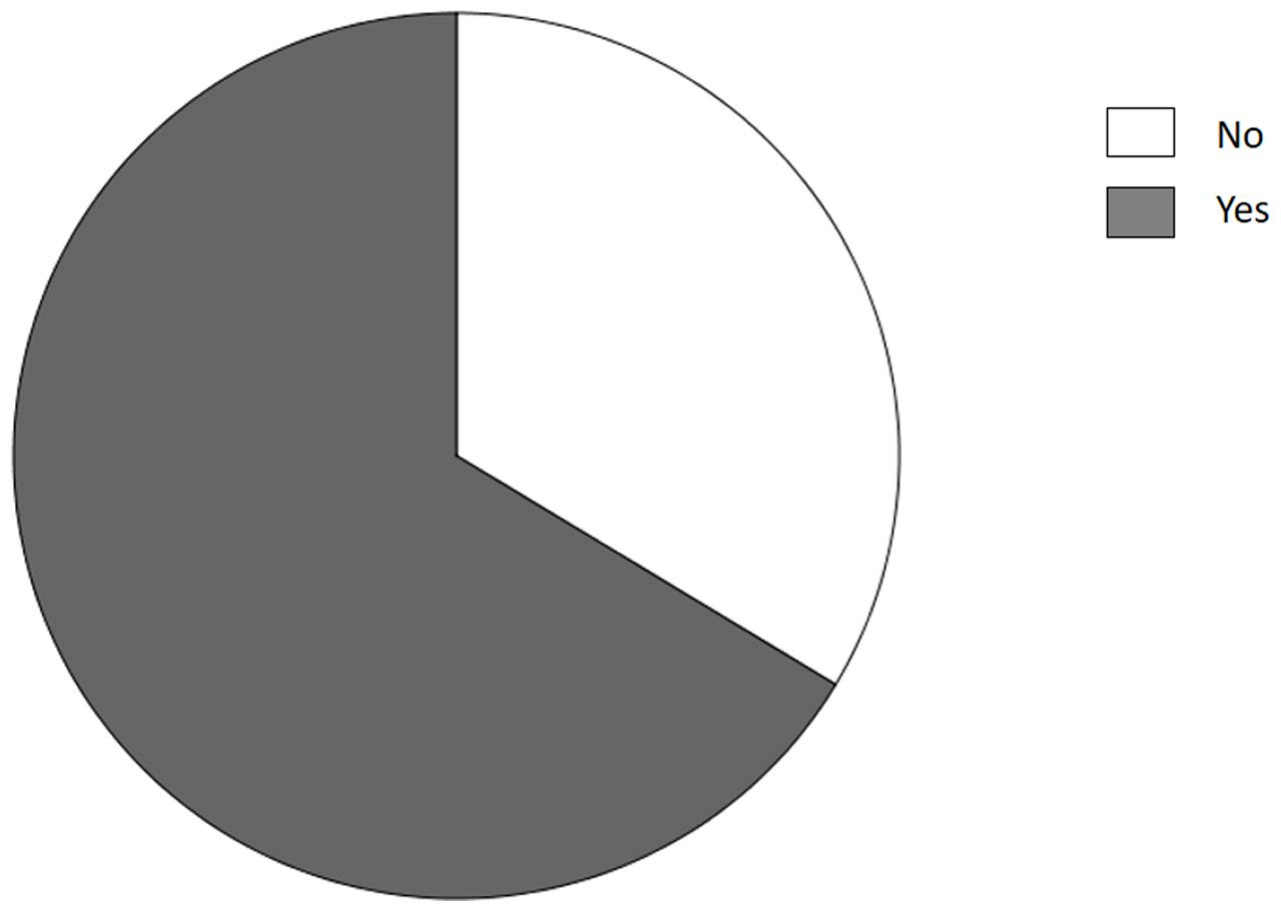
Changes in eating habits after diagnosis of endometriosis.

After the diagnosis of endometriosis, the eating habits changed in 66.4% of the responders (Figure 1).

**Figure 2:**
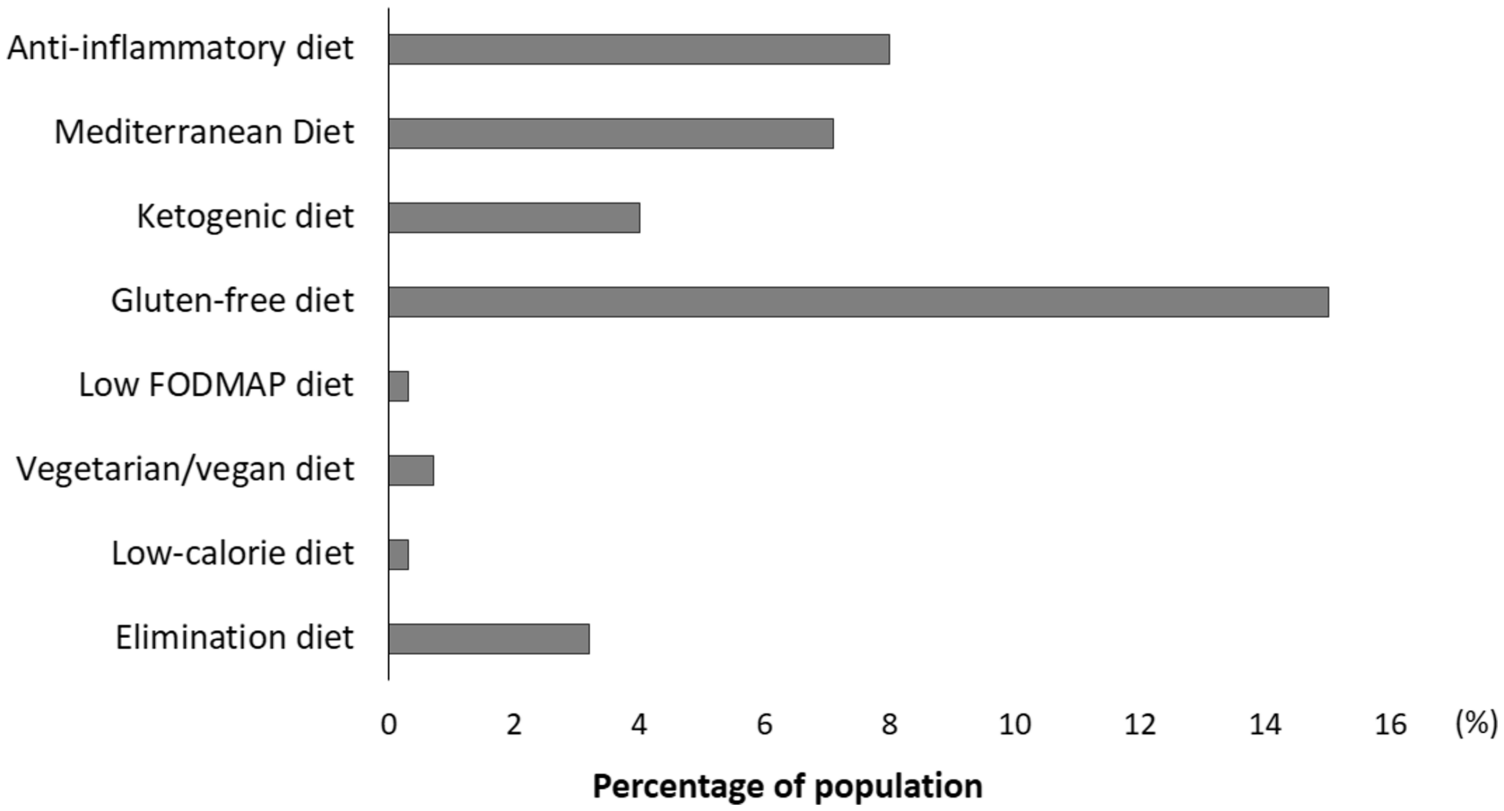
Dietary patterns followed by women with endometriosis.

Some women reported following various dietary patterns after endometriosis diagnosis to improve their health and reduce symptoms (Figure 2). Most of the responders has chosen a gluten-free diet (15%), anti-inflammatory diet (8%), Mediterranean diet (7.1%), ketogenic diet (4%), and other dietary patterns (Figure 2).

**Figure 3:**
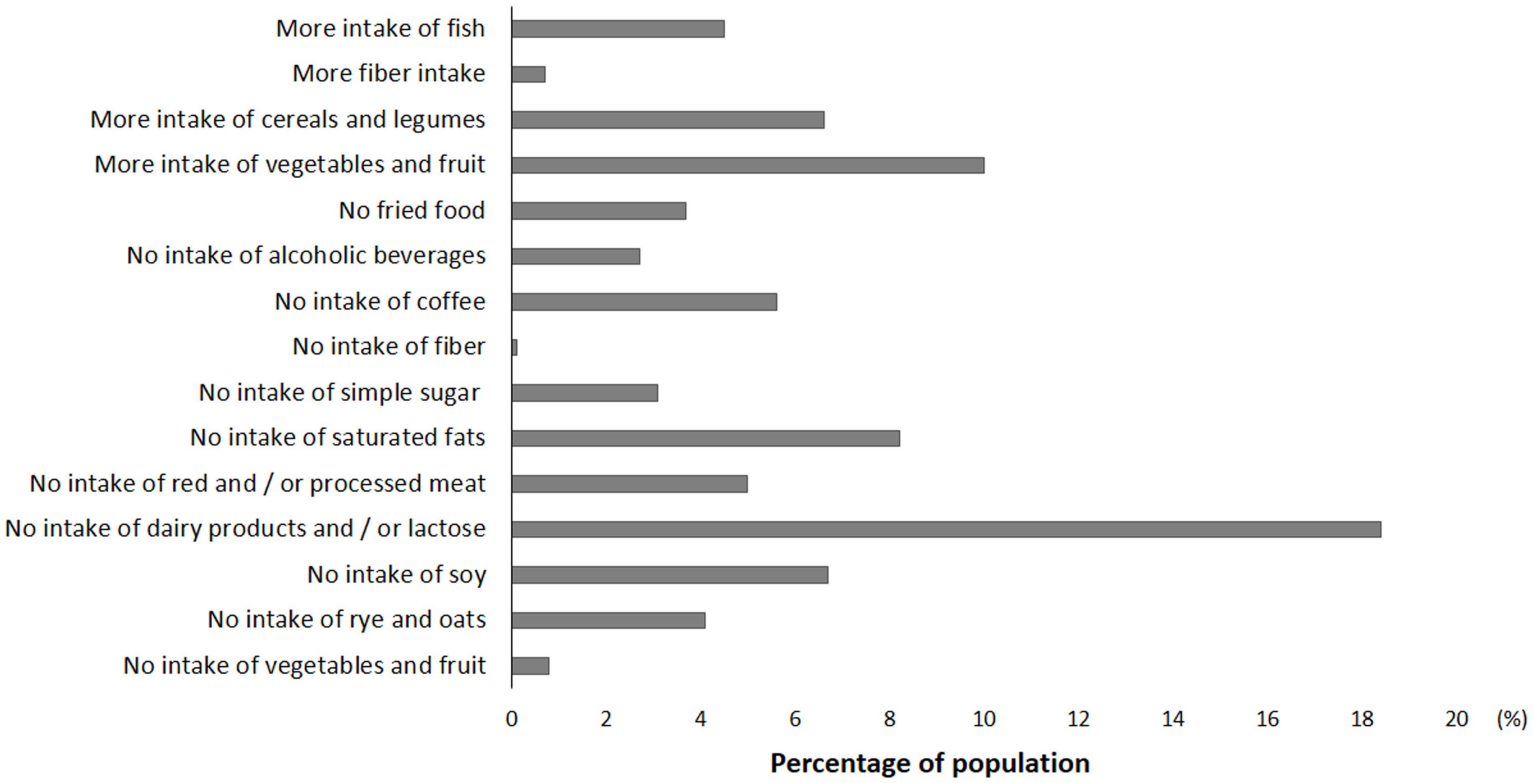
Food choices of women with endometriosis.

Other responders reported a change in eating habits by increasing, or excluding the intake of specific foods or macronutrients or certain cooking methods (Figure 3). Most of the participants excluded dairy products (18.4%), foods with soy (6.7%), and foods with high content of saturated fats (8%) (Figure 3). Other participants instead reported an increase in consumption of vegetables and fruit (10%), cereals and legumes (6.6%), and fish (4.5%).

### 3.2. Impact of endometriosis symptoms on everyday life

**Figure 4:**
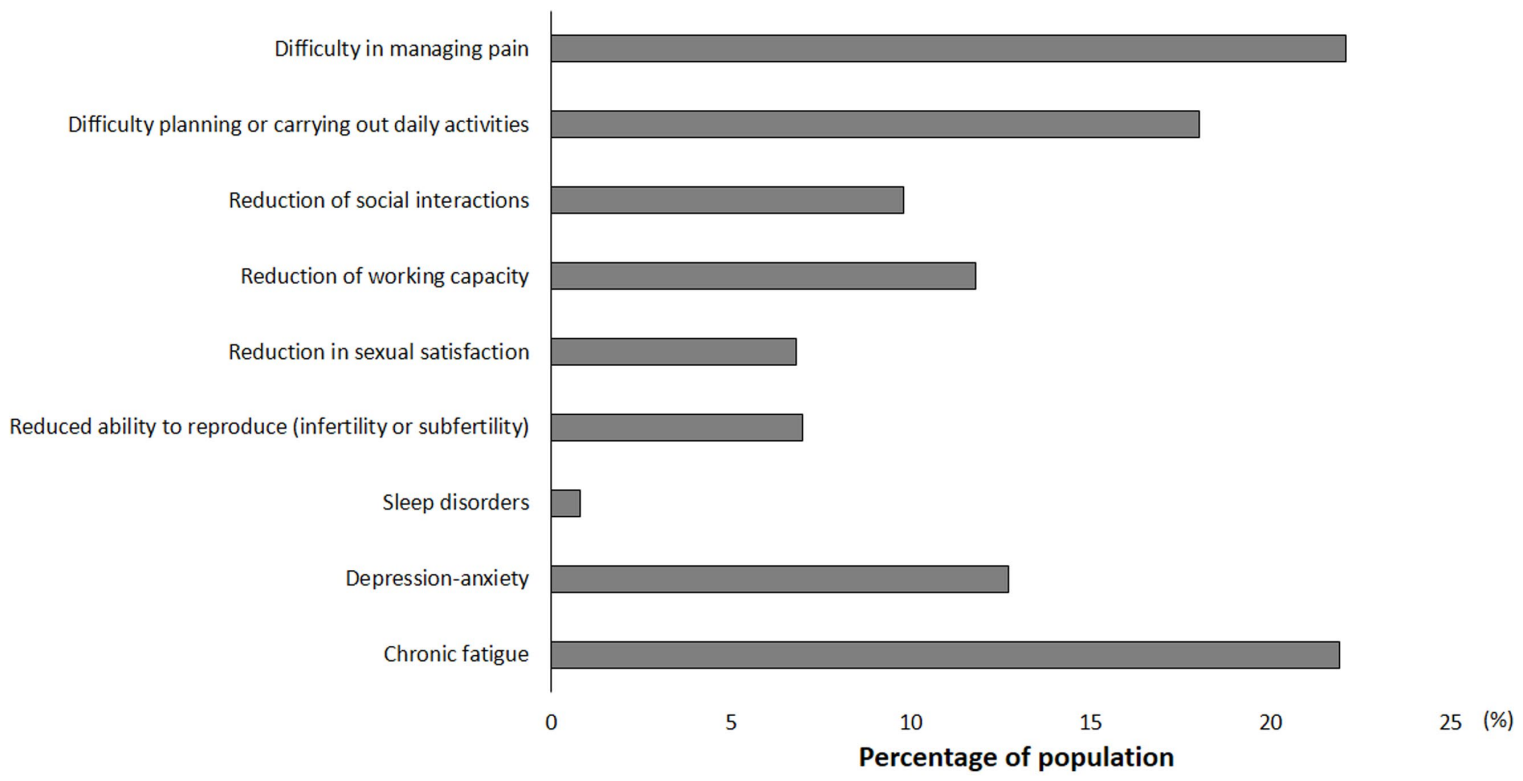
Impact of Endometriosis on everyday Life.

Family and friends tend to minimize endometriosis symptoms for 46% (*n* = 1872) of responders (data not shown). In addition, the everyday Life worsened for 92% (*n* = 3,767) of the women interviewed due to the disease. In particular, women with endometriosis reported difficulty in managing pain and difficulty planning or carrying out daily activities (22%), reduction of working capacity and (12%), and reduction of social interactions (10%) (Figure 4). Furthermore, chronic fatigue and depression-anxiety were reported by 22% and 13% of women with endometriosis, respectively (Figure 4).

### 3.3. Predictors of changes in eating habits and the worsening of the everyday life

In women with endometriosis, Pearson’s correlation showed that changes in eating habits correlated with endometriosis stages (*r* = 0.10, *p* < 0.001), years of symptoms (*r* = 0.37, *p* = 0.018), and changes everyday Life (*r* = 0.14, *p* < 0.001) (data not shown). Moreover, in this population, the worsening of the everyday Life was correlated with educational level (*r* = −0.04, *p* = 0.002), endometriosis stages (*r* = 0.15, *p* < 0.001), years of symptoms (*r* = 0.13, *p* < 0.001), and changes in eating habits (*r* = 0.14, *p* < 0.001) (data not shown).

In the logistic regression analysis, changes in eating habits were found to be associated with endometriosis stages and a decline in everyday life (Table 2). Moreover, changes in everyday life were significantly associated with all variables, including educational level, endometriosis stages, years of symptoms, and changes in eating habits (Table 2).

**Table 2:** Logistic regression analysis – demographic and clinical factors associated with the worsening of everyday life and changes in eating habits.

Dependent variable worsening of the quality of life	*B*	SE	*p*	OR	95% CI	
					LL	UL
Educational level	−0.32	0.12	0.009	0.72	0.57	0.92
Endometriosis stages	0.37	0.05	<0.001	1.45	1.30	1.61
Years of symptoms	0.24	0.04	<0.001	1.27	1.17	1.38
Changes in eating habits	0.96	0.12	<0.001	2.61	2.05	3.32

Dependent variable changes in eating habits*	*B*	SE	*p*	OR	95% CI	
					LL	UL
Endometriosis stages	0.14	0.02	<0.001	1.15	1.09	1.22
Worsening of the quality of life	0.96	0.12	<0.001	2.62	2.06	3.33

*Excluded variables: years of symptoms. *B*, unstandardized coefficient; SE, standard error; OR, odds ratio; CI, confidence interval; LL, lower limit; UL, upper limit.

Thus, we analysed changes in eating habits based on the severity of the disease (Table 3). We found that women with stage IV of endometriosis they had greater adherence to the anti-inflammatory diet (*p* < 0.001) than those in stage I (Table 3). Moreover, in the severe stage, there is a higher prevalence of women who have eliminated the consumption of dairy products and/or lactose (*p* < 0.001), red and/or processed meat (*p* = 0.003), foods with high intake of saturated fats (*p* < 0.001), with simple sugar (*p* = 0.03), and fried foods (*p* = 0.03) than those with minimal stage of endometriosis (stage I) (Table 3). Furthermore, women with stage III and IV reported increased consumption of vegetables and fruit (*p* = 0.04), cereals and legumes (*p* = 0.03), and fish (*p* = 0.02) compared to women with stage II and I of endometriosis (Table 3).

**Table 3:** Dietary models and foods choices among women with endometriosis according to stages of disease.

Variables	Stage I (*n* = 962)	Stage II (*n* = 773)	Stage III (*n* = 831)	Stage IV (*n* = 1,512)	*p*-value
Anti-inflammatory diet	5.3	7.6	7.7	9.7	<0.001
Mediterranean Diet	7.8	7.6	6.9	6.6	0.21
Ketogenic diet	3.3	3.8	4.0	4.6	0.11
Gluten-free diet	10.8	15.3	17.1	15.0	0.007
Low FODMAP diet	0.5	0.4	0.2	0.2	0.14
Vegetarian/vegan diet	0.6	0.8	0.8	0.7	0.79
Low-calorie diet	0.3	0.1	0.4	0.4	0.51
Elimination diet	2.0	3.8	3.6	3.4	0.10
No intake of vegetables and fruit	0.3	0.6	1.2	1.0	0.046
No intake of rye and oats	3.1	4.0	4.3	4.7	0.06
No intake of soy	5.7	6.5	7.6	7.1	0.15
No intake of dairy products and/or lactose	13.0	18.2	20.5	20.7	<0.001
No intake of red and/or processed meat	3.4	4.8	4.7	6.2	0.003
No intake of saturated fats	5.7	7.9	7.9	10.1	<0.001
No intake of simple sugar	2.5	2.5	3.1	3.8	0.035
No intake of fiber	0.2	0.1	0.0	0.2	0.93
No intake of coffee	4.3	6.0	5.9	6.1	0.08
No intake of alcoholic beverages	2.6	3.0	2.5	2.6	0.91
No fried foods	3.0	2.8	3.7	4.4	0.034
More intake of vegetables and fruit	8.6	10.1	9.3	11.3	0.045
More intake of cereals and legumes	5.6	5.8	6.7	7.6	0.032
More fiber intake	0.4	0.6	0.6	0.9	0.20
More intake of fish	3.5	3.6	5.4	5.1	0.029

Data are reported as a percentage (%).

**Figure 5:**
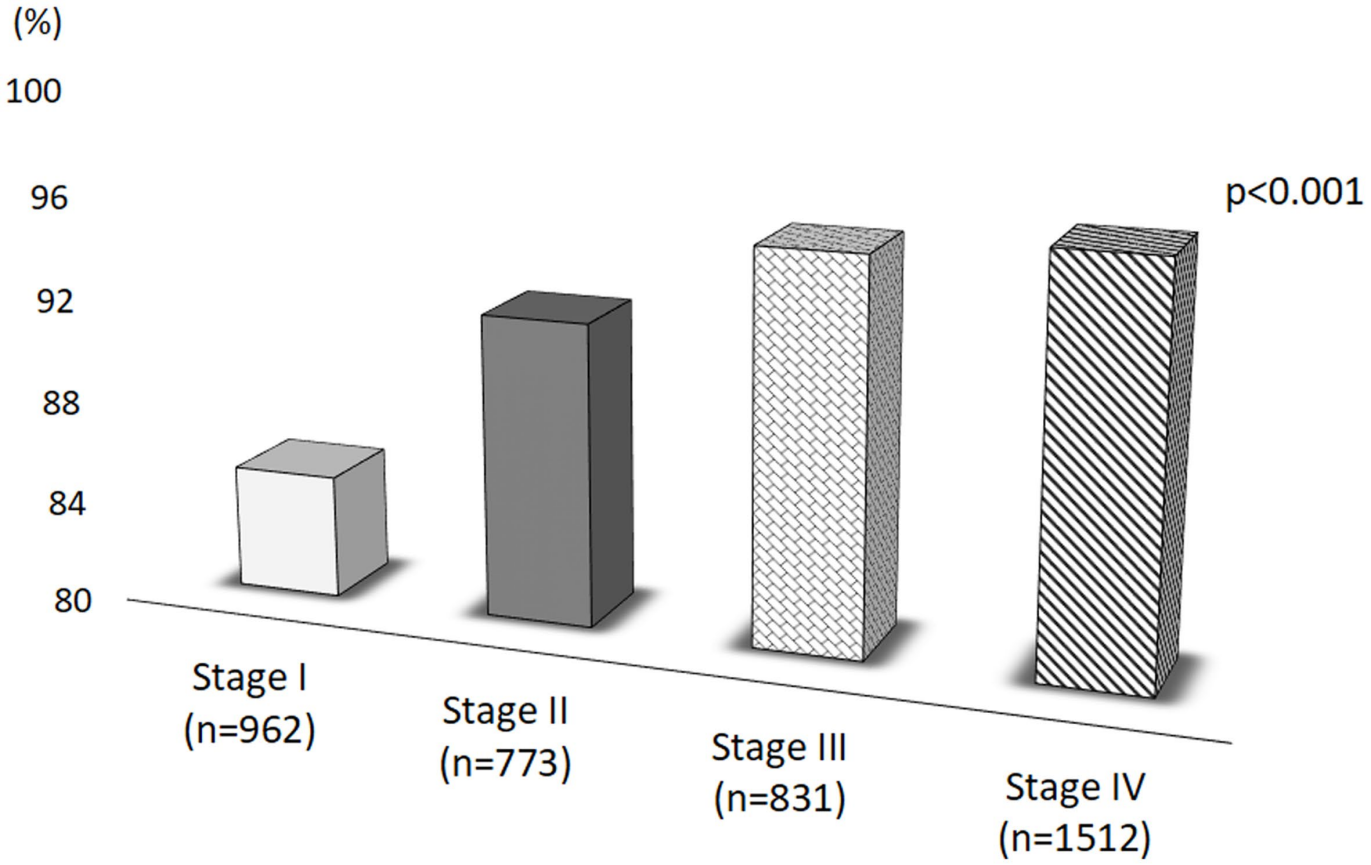
Worsening of everyday Life according to stages of disease.

We also found a higher prevalence of worsening of everyday Life in women with stage III and IV compared to women with stage III and II of endometriosis (Figure 5).

Finally, Table 4 shows the food choices of women with and without worsening of the quality of life due to endometriosis. A higher prevalence of women with worsening of the quality of life chose a gluten-free diet (15%), an anti-inflammatory diet (8%) and a ketogenic diet (4%) than women without worsening of the quality of life. In addition, a higher prevalence of women with worsening of the quality of life eliminated the consumption of dairy products and/or lactose (19%), red and/or processed meat (5%), foods with high intake of saturated fats (8%), and fried foods (4%) than those without worsening of the quality of life (Table 4). Furthermore, women with worsening of the quality of life reported increased consumption of vegetables and fruit (10%), cereals and legumes (7%), and fish (5%) compared to women worsening of the quality of life due to endometriosis (Table 4).

**Table 4:** Dietary models and Foods choices of women with endometriosis according to worsening of everyday Life.

Variables	Women without a worsening of the everyday life (*n* = 311)	Women with a worsening of the everyday life (*n* = 3,642)	*p*-value
Anti-inflammatory diet	4.8	8.1	0.037
Mediterranean Diet	4.5	7.4	0.06
Ketogenic diet	1.9	4.2	0.05
Gluten-free diet	9.0	14.9	0.003
Low FODMAP diet	0.6	0.3	0.26
Vegetarian/vegan diet	0.3	0.8	0.72
Low-calorie diet	0.3	0.3	1
Elimination diet	1.3	3.3	0.06
No intake of vegetables and fruit	0.3	0.8	0.51
No intake of rye and oats	1.3	4.4	0.007
No intake of soy	3.2	7.0	0.007
No intake of dairy products and/or lactose	8.4	19.2	<0.001
No intake of red and/or processed meat	1.9	5.2	0.009
No intake of saturated fats	5.1	8.4	0.041
No intake of simple sugar	1.3	3.3	0.06
No intake of fiber	0.0	0.2	1
No intake of coffee	2.3	5.9	0.005
No intake of alcoholic beverages	0.6	2.8	0.016
No fried foods	1.3	3.8	0.017
More intake of vegetables and fruit	5.5	10.4	0.004
More intake of cereals and legumes	3.5	6.9	0.023
More fiber intake	0.0	0.7	0.26
More intake of fish	1.9	4.7	0.022

Data are reported as a percentage (%).

## 4. Discussion

The aim of this study was to investigate the potential effects of the disease on dietary habits and daily activities in women following an endometriosis diagnosis. The large cohort of 4,078 participants provides a robust basis for data analysis. The majority of women falling between the ages of 36 and 45 may reflect the typical age of diagnosis or when women actively seek medical assistance (4, 5). The prevalence of 37% of women with severe stage (stage IV) is a significant representation of those facing the most challenging issues associated with endometriosis (4, 5, 10–12). The delay in diagnosis exceeding 7 years for 39% of the participants is concerning and highlights the importance of timely and accurate diagnosis. Several studies have reported similar diagnostic delays to those observed in our population, with an average time from the onset of initial symptoms to a definitive diagnosis ranging from 4.4 years in the United States to 10.4 years in Germany (41, 42). The primary reasons for such delays include intermittent contraceptive use, self-treatment of pain with over-the-counter analgesics, and misdiagnosis.

The fact that 66.4% of women reported making changes in dietary habits after an endometriosis diagnosis suggests a significant impact of the disease on women’s perception of nutrition and health (14). The reported dietary choices, such as gluten-free, anti-inflammatory, Mediterranean, and ketogenic diets, indicate that women are exploring diverse dietary approaches to address symptoms and improve their quality of life. The analysis of dietary choices based on the disease stage revealed some significant differences. Women with severe endometriosis (stage IV) appear to more frequently follow an anti-inflammatory diet while eliminating foods high in saturated fats and simple sugars. These findings may indicate an attempt to manage inflammation associated with severe endometriosis. Simultaneously, greater adherence to an anti-inflammatory diet might reflect a response to symptom severity and the need to address inflammatory processes. On the other hand, differences in dietary choices could also be influenced by women’s increased awareness of the effect of nutrition on health. The findings of our study are in line with current understanding in the field.

A retrospective Italian study examined the effects of a gluten-free diet on endometriosis-associated symptoms (23). At the 12-month follow-up, 52% of patients re-ported statistically significant improvements in pain compared to baseline (23). Interestingly, approximately 30% of patients did not adhere to the gluten-free diet (23). A gluten-free diet may be beneficial for patients experiencing gastrointestinal-related abdominal pain, constipation, bloating, and suspected visceral hypersensitivity. However, adherence to such diets may be compromised due to financial constraints and inherent difficulties. Our study revealed that the anti-inflammatory diet was more commonly followed by women with severe-stage disease. Although popular, there is currently insufficient scientific evidence to support the role of this diet in managing endometriosis.

A single-arm study conducted in Australia examined the effects of the Mediterranean diet on endometriosis-associated pain (43). Patients adhered to a specific diet that included fruits, fresh vegetables, fatty fish, white meat, soy products, whole grain products, magnesium-rich foods, and extra virgin olive oil. Furthermore, the Mediterranean diet includes the consumption of many spices. Significant pain relief, including improvements in general pain, dysmenorrhea, dyschezia, dyspareunia, and overall condition, was observed (43). The Mediterranean diet may alleviate endometriosis-related pain through synergistic mechanisms. Extra virgin olive oil and fish have been shown to exert anti-inflammatory effects (44, 45). Oleocanthal, found in extra-virgin olive oil, exhibits a molecular structure similar to that of ibuprofen and exerts a cyclooxygenase inhibition effect via the same mechanism (46). Furthermore, the antioxidant effects, abundant fiber content, and magnesium present in the Mediterranean diet may have positive effects on pelvic pain and inflammation (47, 48). Moreover, some spices such as onions, rosemary, chili peppers, ginger, turmeric, garlic, are commonly incorporated into the Mediterranean Diet and antinflammatory pattern. Recent preclinical and clinical studies have substantiated the effectiveness of these spices and their bioactive compounds in preventing and mitigating various chronic diseases, including arthritis, asthma, cancer, neurodegenerative disorders, and cardiovascular conditions (49, 50). These spices hold the potential to alleviate the inflammatory effects associated with endometriosis.

Our findings align with previous studies in which female participants reported that avoiding or limiting a wide range of nutrients, including dairy, gluten, soy, sugar, and coffee, helped alleviate their symptoms, while adding fruits or vegetables proved beneficial (51). Several epidemiological studies have associated a high consumption of fruits (52), omega-3 fatty acids (15), and dairy during adolescence (53) with a reduced risk of developing endometriosis. Conversely, high consumption of trans-unsaturated fats (15), red meat (16), and alcohol (54) have been associated with an increased risk, although it remains unclear whether these factors also influence the symptoms of diagnosed endometriosis. Some participants in our study also adopted a ketogenic diet, which is high in fats, moderate in proteins, and very low in carbohydrates. This diet promotes the production of endogenous ketones as an alternative metabolic fuel source (55). Preclinical studies have demonstrated positive effects of the ketogenic diet on oxidative stress markers and inflammation, which are relevant to endometriosis (56–59). However, there is currently insufficient scientific evidence to support the use of this dietary protocol for endometriosis. A prospective controlled study demonstrated the anti-inflammatory effects of a nutraceutical containing vitamin B3, omega-3/6, quercetin, calcium salt, 5-methyltetrahydrofolate, parthenium, and turmeric in women with endometriosis (19). This study revealed significant reductions in pain symptoms and serum levels of CA-125, PGE2, and 17β-estradiol in the group treated with the nutraceutical (19). A small proportion (5%) of women in our questionnaire reported using this nutraceutical for pain relief. However, due to the unclear long-term safety of dietary antioxidant supplementation beyond 6 months, prolonged use cannot be recommended (60, 61).

Importantly, the correlation between dietary habit changes, endometriosis stage, and symptom duration suggests that some dietary adjustments may be adaptive responses to symptom severity or disease progression. The negative effect of the disease on women’s everyday life is evident in the study’s results and is consistent with findings from other scientific studies (7, 8, 10, 11, 15). Family and friends’ minimizing attitude toward symptoms can contribute to increased emotional burden for women facing misunderstanding. The worsening of quality of life, as indicated by difficulties in managing pain and planning daily activities, highlights a range of physical and psychological challenges that women must confront, which aligns with observations in other studies (7, 8, 10, 11, 15). The prevalence of 22% for chronic fatigue and 13% for depression-anxiety underscores the importance of a comprehensive and integrated approach to endometriosis management.

The results from our study underscore the necessity of providing specific nutritional guidance to women with endometriosis. Currently, there are no established nutritional guidelines for this clinical condition, highlighting the need for clinical trials to identify the optimal nutritional strategies for alleviating endometriosis symptoms.

Our study possesses several strengths, including the use of an online survey, which facilitated the rapid recruitment of a sizeable sample of women. The participants exhibited diverse age ranges, educational backgrounds, and endometriosis stages according to the ASRM classification. However, our study also has limitations. Firstly, it is a cross-sectional study. Secondly, we relied on self-reported data and did not employ a food frequency questionnaire or food diary to assess macro- and micro-nutrient deficiencies. or explore the impact of these conditions using validated questionnaires (38). In addition, we categorized the age of the patients; however, we did not establish a maximum age cutoff within the scope of this study. Furthermore, we did not investigate anthropometric parameters or the effects of endometriosis diagnosis on the risk of malnutrition. Additionally, there may be a selection bias as the survey participants were members of the Italian endometriosis association, potentially indicating a higher level of health awareness compared to the general population. Nonetheless, despite these limitations, our study generates hypotheses for future investigations. Currently, there is limited research available on the effects of dietary modifications on endometriosis-associated symptoms, with only a small number of studies (19, 27, 41–43) addressing this topic. However, several dietary approaches have been proposed as potential strategies to mitigate the progression of endometriosis and improve clinical symptoms. Nonetheless, further studies are needed to investigate the efficacy of these dietary interventions.

## 5. Conclusion

In conclusion, our study provides evidence that dietary changes are commonly employed by women with endometriosis as a self-management tool. These women are utilizing various dietary modifications to alleviate their endometriosis symptoms. However, it remains unknown which specific dietary interventions are effective for women with different types of endometriosis or specific individual characteristics. The findings of our study highlight the importance of referring patients to nutritional counselling to prevent nutritional deficiencies. It is crucial to educate women about the objectives and rationale of the dietary intervention and to provide guidance on which nutrients to include or avoid. Future clinical trials investigating the efficacy of specific diets for women with endometriosis will help tailor individualized dietary approaches for optimal patient outcomes.

## Data availability statement

The raw data supporting the conclusions of this article will be made available by the authors, without undue reservation.

## Ethics statement

The studies involving humans were approved by Local Ethics Committee in the Calabria Region Central Area. The studies were conducted in accordance with the local legislation and institutional requirements. The participants provided their written informed consent to participate in this study.

## Author contributions

EM: Conceptualization, Writing – review & editing, Data curation, Methodology, Writing – original draft. ET: Conceptualization, Methodology, Writing – original draft. SM: Writing – original draft, Data curation. YF: Conceptualization, Formal analysis, Writing – review & editing. AA: Methodology, Investigation, Writing – original draft. MA: Methodology, Investigation, Writing – original draft. TM: Supervision, Writing – review & editing. AP: Supervision, Writing – review & editing.
