# Supplementary material for: The impact of endometriosis on dietary choices and activities of everyday life: a cross-sectional study
Source: Front Nutr. 2023 Sep 22;10:1273976. doi: 10.3389/fnut.2023.1273976 (PMC10559972; doi:10.3389/fnut.2023.1273976)
Supplement: Supplementary file 1 [file Data_Sheet_1.PDF]

Supplementary Table S1

SURVEY ON LIFESTYLE CHANGES IN ITALIAN WOMEN WITH ENDOMETRIOSIS

Dear Participant,

We are conducting a survey to investigate the LIFESTYLE CHANGES IN ITALIAN WOMEN WITH ENDOMETRIOSIS. The compilation of this questionnaire is anonymous and takes a few minutes. Please click on the answer that you consider closest to your opinion. The answers will remain confidential and will be used exclusively for statistical purposes in compliance with current legislation on the confidentiality of personal data. The data collected will be processed in compliance with the privacy legislation (Information for the processing of personal data, pursuant to Legislative Decree 196/2003 coordinated with Legislative Decree 101/2018).

Thanks for the precious collaboration.

How old are you?

- 18 - 25 years
- 26 - 35 years
- 36 - 45 years
- ≥ 45 years

Education Level:

- High School Diploma
- Bachelor's Degree

What stage of endometriosis do you have?

- Stage I
- Stage II
- Stage III
- Stage IV
- I don't know my current stage

How long have you experienced symptoms of endometriosis?

____

How long did you wait to receive a diagnosis of endometriosis?

- < 1 year
- 1 year - 3 years
- 4 years - 6 years
- ≥ 7 years

What medications are you taking for endometriosis?

Have you noticed any changes in your mouth after taking these medications?

- Yes
- No

Do you suspect you have periodontitis?

- Yes
- No
- I don't know

How would you describe the health of your teeth and gums?

- Insufficient
- Sufficient
- Good
- Very Good
- Excellent
- I don't know

Have you undergone root planing sessions in the past?

- Yes
- No

Have you noticed tooth mobility not caused by trauma?

- Yes
- No

Do you suffer from recurrent canker sores, herpes, or other oral lesions?

- Yes
- No
- I don't know

Do you experience a burning sensation on your tongue or in your entire oral cavity?

- Yes
- No

**Have you noticed changes on the surface of your tongue since suffering from endometriosis?**<br><br>- Yes<br>- No
**Since suffering from endometriosis, have you noticed if your oral problems (gingivitis, periodontitis, gum bleeding, canker sores, dry mouth) have worsened or if they have not improved despite treatment?**<br><br>- Yes<br>- No<br>- I don't know
**If you answered YES to the previous question, what advice or treatment have you received for your oral problems? ____________________**
**Do you have autoimmune diseases (Sjögren's syndrome, rheumatic diseases, etc.)?**<br><br>- Yes<br>- No<br>- I don't know
**If you answered YES to the previous question, please indicate which autoimmune diseases you have ________________**
**Is your dentist and/or dental hygienist aware of your condition?**<br><br>- Yes<br>- No
**Do your family/friends tend to minimize your symptoms?**<br><br>- Yes<br>- No<br>- I don't know
**Has your quality of life been worsened by your condition?**<br><br>- Yes<br>- No<br>- I don't know
**In what ways has your condition influenced your quality of life?**<br><br>- chronic fatigue<br>- depression and anxiety<br>- sleep disorders<br>- reduced fertility or subfertility

- decreased sexual satisfaction
- reduced work capacity
- diminished social interactions
- difficulties in daily activity
- pain management challenges
- No way

Before receiving the diagnosis, what was your consumption of fruits and vegetables?

- I don't consume them or rarely
- 1-2 servings per day
- 3-4 servings per day
- At least 5 servings or more per day

Before receiving the diagnosis, what was your alcohol consumption?

- Never or rarely
- 1-3 units per week
- 4-6 units per week
- Every day, 1 or more per day

Have you changed your diet following the diagnosis of endometriosis?

- Yes
- No

How has your diet changed?

- Anti-inflammatory diet
- Mediterranean Diet
- Ketogenic diet
- Gluten-free diet
- Low FODMAP diet
- Vegetarian/vegan diet
- Low-calorie diet
- Elimination diet
- No intake of vegetables and fruit
- No intake of rye and oats
- No intake of soy
- No intake of dairy products and / or lactose
- No intake of red and / or processed meat
- No intake of saturated fats
- No intake of simple sugar
- No intake of fiber
- No intake of coffee
- No intake of alcoholic beverages
- No fried foods
- More intake of vegetables and fruit
- More intake of cereals and legumes

- More fiber intake
- More intake of fish

Before the diagnosis, how many minutes of physical activity did you engage in per week (e.g., gym workouts like pilates, yoga, aerobic activities, brisk walking, or running)?

- 5 or more times a week
- 3-4 times a week
- 1-2 times a week
- Less frequently
- I have not engaged in sports-related physical activity

Are you aware that endometriosis can indirectly be related to pelvic floor muscle disorders?

- Yes
- No

During sexual intercourse, if you ever experienced pain, how was it presented?

- At the vaginal entrance
- Deeply
- In both cases
- I do not have sexual intercourse

If you have undergone surgery for endometriosis and suffered from pain during intercourse, has this symptom been modified by the intervention?

- Yes
- No
- I don't know

Are you aware that there is physical therapy and rehabilitation for symptoms related to pelvic floor dysfunction?

- Yes
- No

Add any additional considerations or data that you believe are useful ___________
